# Tailoring interventions for social isolation among older persons during the COVID-19 pandemic: challenges and pathways to healthcare equity

**DOI:** 10.1186/s12939-020-01360-8

**Published:** 2021-01-08

**Authors:** Lise Dassieu, Nadia Sourial

**Affiliations:** 1 Research Center of the Centre Hospitalier de l’Université de Montréal, 850 St Denis Street, Saint Antoine Tower, Office S01.135, H2X 0A9 Montréal, Quebec Canada; 2 Department of Family and Emergency Medicine, Faculty of Medicine, Université de Montréal, Montréal, Quebec Canada

**Keywords:** Social isolation, Older persons, COVID-19, Equity, Person-centered care

## Abstract

**Background:**

Social isolation among older adults raises major issues for equity in healthcare in the context of the COVID-19 pandemic.

**Main text:**

This commentary describes current challenges in preventing social isolation among older adults and proposes pathways to develop inclusive approaches to intervention in this vulnerable population. Building interventions that take account of structural inequities among older persons, as well as their subjective experiences, expectations and perspectives, appears fundamental to improve their health and quality of life in pandemic and post-pandemic contexts.

**Conclusions:**

We argue that equity-based and person-centered approaches are critical to counter the negative outcomes of social isolation in the vulnerable older population.

## Background

The COVID-19 pandemic has led to many deleterious consequences for older persons in the world. In addition to higher risks of COVID-19-related complications and mortality, this population has been disproportionately impacted by preventative measures such as physical distancing, aimed at reducing the risk of infection. This situation has resulted in increased social isolation among older adults in many countries [1, 2]. The first wave of the COVID-19 pandemic has served to bring to the foreground the numerous negative health outcomes associated with social isolation in older adults, at the physical and psychological levels [3]. Indeed, social isolation is a well-documented risk factor for poor quality of life, physical and mental health problems, and premature mortality [4, 5], with a level of risk comparable to smoking, obesity and hypertension. With the second wave of the pandemic well underway and the prospect of further isolation in the coming winter months, the need to better screen and support this vulnerable population is at a critical point.

## Main text

### New challenges to assessing and preventing social isolation among older adults

Some tools have been developed before and during the pandemic’s first wave to identify and care for isolated older persons in the community [6–8]. Pre-pandemic interventions to prevent social isolation include standardized assessment scales [9–11] and diverse types of in-person interventions (e.g. leisure-based group meetings, individual encounters, service provision…), as well as some online interventions [7, 8, 12]. However, literature shows that not every older adult is likely to benefit from these interventions [7, 12]; and pre-pandemic intervention frameworks may not be suitable to identify and address the new, multifaceted realities of older adults’ isolation during the current pandemic. Some online and/or telephone-based interventions have started to be developed to maintain contact with older adults during COVID-19 first wave [13] or to assess their health care and social support needs [6]. The creation and implementation of such new screening and prevention tools need to take into account the variability in older adults’ situations and perspectives, as these dimensions may impact experiences of social isolation. These considerations should be addressed to prepare for future COVID-19 waves and ensure that care responses meet the needs of older persons facing this unprecedented crisis.

### Diversity in the older population: the burden of social inequities

Psychosocial and healthcare interventions need to be adaptable to the diverse situations that can lead to social isolation in older persons. Social isolation may result from a number of different pathways, with various combinations of physical, mental, socio-economic and socio-cultural factors. Several recent studies showed that structural inequities, which influence housing conditions, the extent of social networks, and access to online technologies, can increase the overall negative impacts of the COVID-19 pandemic among older adults [14, 15] and affect their social isolation [16].

Indeed, inequities and discriminations are important risk factors for overall poor health and decreased life expectancy [17]. Intersectional discrimination, that is discrimination across an intermingling of factors such as socio-economic status, gender and race, can also be combined with age-based discrimination, resulting in worsened health outcomes for older persons from specific social groups [18].

To foster equity in responses preventing social isolation among older adults, interventions need to target and adapt to the specific challenges of deprived social groups and racial minorities. Co-constructing interventions with persons from these groups could help foster inclusiveness and appropriateness. Building and implementing equity-based approaches to intervention requires the use of techniques to offer a safe space allowing every older person’s engagement. To provide optimal conditions, it is essential to make everyone comfortable and welcome, pay attention to unequal power relationships and consider needs for confidentiality. For example, culture sensitivity may be improved by recruiting professionals or volunteers of the same native language or culture as the targeted older adults [19]. Recognizing the importance of the social determinants of health and the specific psychological realities of older adults from deprived social groups and minorities appears crucial both in the process of co-constructing participatory interventions and for implementing them. Indeed, care practices and programs need to be tailored to older persons’ personal histories (e.g. experiences of trauma, violence) and social contexts (e.g. cultural and familial characteristics, financial barriers, housing conditions). Some resources to foster equity-oriented and trauma-informed care have been successfully implemented in primary care settings [20]. They offer practical tools to educate clinicians and reorganize health care services on the basis of equity [19]. Such inspiring intervention frameworks could be transferred and adapted to interventions preventing social isolation among older adults during the pandemic. This could help raise providers’ awareness of the importance of addressing systemic inequities and respecting socio-cultural diversity during their work with older persons.

### Perceived isolation: considering older persons’ subjective experiences

Another major challenge for intervention is to identify new and unusual forms of isolation resulting from older adults’ experiences and feelings during the pandemic. Physical distancing measures have not only affected older adults that were already socially isolated or vulnerable to isolation, but have also plunged many into new enduring states of isolation. Some persons that were usually considered to be protected against isolation by a sufficient social network, such as those with their children living nearby, may experience a new sense of social isolation due to physical distancing measures, though they do not fit traditional definitions of isolation. This poses an important challenge to isolation screening as most of the existing assessment tools are based on standard validated scales screening variables such as frequency of contacts and size of social network. Such quantitative evaluation scales, however, have already been shown to imperfectly capture the diversity of individual experiences of isolation [7]. The conceptualization of social isolation may therefore need to move beyond pre-pandemic intervention frameworks, to include more subjective or less apparent forms of isolation for individuals. Differentiating isolation from loneliness also appears crucial to consider in this context [16].

Older persons’ perceived isolation, which has been shown to be associated with increased risk of anxiety and depression [21], could be a useful indicator to screen for early stages of isolation and prevent risks of aggravation. Screening interventions should therefore target a more qualitative and person-centered approach considering older adults’ perspectives, especially their subjective experience and sense of isolation and loneliness. This goal may be achieved through the use of open conversations in addition to standardized tools measuring objective variables such as social connectedness. Involving older persons from diverse social and cultural backgrounds in defining the meaning and extent of social isolation in the current perspective of medium/long-term physical distancing measures would provide the insight needed to develop appropriately tailored interventions when needed.

## Conclusions: Addressing social isolation through equitable and person-centered care

With social distancing and other COVID-19 public health measures likely to continue for many more months to come, identifying and supporting the increasing number of older adults at risk or suffering from social isolation is critically needed. In order to both reduce risks of serious COVID-19 complications and prevent social isolation in this population, it is crucial to acknowledge social inequities in older people’s experiences and challenges, as well as their diverse perspectives on social isolation. Special attention should be given to the most vulnerable and deprived social groups. Interventions need to be adapted to be more person-centered, more attuned to the diverse pathways leading to states of isolation and more mindful of the importance of perceived social isolation as an early sign of increased vulnerability. Building interventions on these principles could help foster equity in healthcare for older persons and prevent the adverse effects of social isolation on physical and mental health outcomes.

## Data Availability

Not applicable.
